# The Role of the Pharmacist in a Patient’s Care for Individuals Undergoing Anticoagulant Therapy: A Case Report

**DOI:** 10.3390/life14080986

**Published:** 2024-08-07

**Authors:** Ana M. Sáez-Benito, Loreto Sáez-Benito, María Salazar, Rosa Magallón, Nuria Berenguer

**Affiliations:** 1 Faculty of Health Science, San Jorge University, 50830 Villanueva de Gállego, Spain; lsaezbenito@usj.es (L.S.-B.); msalazarp@usj.es (M.S.); nberenguer@usj.es (N.B.); 2 Aragonese Primary Care Research Group (GAIAP), Institute for Health Research Aragón (IIS Aragón), 50009 Zaragoza, Spain; rosamaga@unizar.es

**Keywords:** vitamin K antagonists, pharmaceutical intervention, pharmacogenetics, SAMe-TT2R2, time in therapeutic range

## Abstract

Achieving clinical effectiveness with vitamin K antagonists (VKAs) requires a Time in Therapeutic Range (TTR) above 65%. TTR is influenced by genetics (CYP2C9, VKORC1, CYP4F2), treatment adherence, and knowledge. The SAMe-TT2R2 algorithm is used to assess VKA treatment suitability. In this case report, SAMe-TT2R2 and pharmacogenetic analysis were used to improve oral anticoagulant management in a patient with poor control of INR. An 84-year-old, obese male with atrial fibrillation, undergoing acenocoumarol therapy, had a suboptimal TTR. An assessment with the SAMe-TT2R2 algorithm indicated a favorable profile for VKA use. An educational intervention on vitamin K-rich foods was conducted, and his physician was informed about the interaction between omeprazole and acenocoumarol, recommending its replacement with pantoprazole. This intervention was accepted by the physician and, three months post-intervention, the patient’s TTR improved to 100%. Poor adherence and limited knowledge contributed to treatment failures in patients with a good VKA profile. Pharmaceutical interventions significantly improved TTR management. Patients with favorable genetic and clinical profiles could achieve adequate control of their anticoagulant medication through these interventions. Predictive tools may help select patients who can effectively and safely use VKAs through pharmaceutical interventions.

## 1. Introduction

The literature demonstrates that to achieve optimal clinical effectiveness with vitamin K antagonist (VKA) coumarin anticoagulants, it is necessary to attain a Time in Therapeutic Range (TTR) greater than 65–70% [1,2]. One of the major limitations in managing VKA drugs is the difficulty, in certain patients, of achieving a stable International Normalized Ratio (INR), as some inherent, unmodifiable conditions in patients confer intrinsic difficulties in achieving that stability. This is particularly evident in the case of genetic variables, especially mutations in the CYP2C9, VKORC1, and CYP4F2 genes, which are associated with the pharmacokinetics and pharmacodynamics of VKAs and are directly linked to intra- and inter-individual variability. The CYP2C9 gene encodes the enzyme responsible for metabolizing the drug; the VKORC1 gene encodes the enzyme involved in the conversion of inactive vitamin K (hepatic reserve or diet) into its active form; the CYP4F2 gene encodes the enzyme vitamin K1 oxidase, which is a metabolizing enzyme for vitamin K [3].

However, it has also been demonstrated that certain clinical factors are associated with a greater difficulty in achieving a stable INR. An example of this is the SAMe-TT2R2 algorithm, which correlates the TTR with factors influencing the anticoagulant effect of VKAs and associates a score of 2 or higher, with increased difficulty in VKA management, for a given patient [4].

The SAMe-TT2R2 algorithm includes various factors affecting the anticoagulation effects of VKAs. These factors are gender (1 point), age younger than 60 years (1 point), individual medical history (1 point), medication interactions (1 point), tobacco use (2 points), and race (2 points). The scoring for each variable is provided within the parentheses. The maximum achievable score is 8 points. If the total score is equal or greater than 2, there is a risk of poor anticoagulation control with VKAs. On the contrary, if the patient scores 0 or 1, they are suitable for anticoagulant treatment with VKAs [4].

The literature also indicates that other oral therapeutic alternatives, such as direct oral anticoagulants (DOACs), may prove to be more effective and cost-effective [5,6], despite the extensive use and lower cost of VKAs. There are two types of DOACs, reversible direct thrombin inhibitors (such as dabigatran) and direct inhibitors of factor Xa (such as edoxaban, rivaroxaban, and apixaban). Both are administered at fixed doses and present a wider therapeutic margin than VKAs [7]. However, even though information in this regard is more limited, it has been demonstrated that when the TTR is stable, the superiority of DOACs is not as evident [8,9].

Multiple experiences have demonstrated that pharmaceutical interventions focused on adherence, knowledge, addressing medication interactions, and diet, etc., in the management of vitamin K antagonist (VKA) drugs, can improve the Time in Therapeutic Range (TTR) [10,11,12]. Therefore, it might be considered that by identifying patients who intrinsically do not have issues with VKA medication management, as they have a favorable genetic predisposition and have a SAMe-TT2R2 score < 2, they could be excellent candidates for achieving a TTR > 70% through pharmaceutical interventions aimed at optimizing these drugs.

## 2. Case Description

An 84-year-old, obese (BMI 31), male patient is undergoing treatment with acenocoumarol (VKA) due to atrial fibrillation. The patient’s baseline characteristics are shown in Table 1. The patient is a candidate for a medication review as he initially presented with a TTR of 56.1% (less than 70%), potentially putting him at risk of adverse effects and/or therapeutic ineffectiveness with VKA treatment.

The pharmacist performs the following interventions (Table 1).

Following this procedure, during the first visit, the baseline variables were collected (Table 2), and the clinical and genetic factors of the patient that could be related to difficulties in achieving a stable INR were analyzed. The SAMe-TT2R2 score was calculated, and the patient scored 2 points. Therefore, based on the demographic, clinical, and pharmacological treatment characteristics, treatment with acenocoumarol did not appear to pose potential problems for this patient. The patient did not exhibit significant genetic limitations for proper INR control (Table 3).

At the second visit, the pharmacist conducted an individualized intervention based on the patient’s characteristics (knowledge, adherence, medication interactions, and diet) (Table 4). Firstly, adherence was assessed using the Morinsky–Green validated test [13], a computerized electronic prescription from the community pharmacy, and an individualized interview with the patient. Unintentional non-adherence was identified. The patient was on multiple medications and frequently forgot many drugs in terms of his regular medication (allopurinol and pregabalin were taken on demand and at a lower dose than prescribed). He was unaware of the correct use of inhalers for his COPD, contributing to a lack of adherence. The patient exhibited intermittent forgetfulness while using acenocoumarol and, additionally, demonstrated limited understanding of this medication, scoring 3 on the “Questionnaire to assess the patient´s knowledge on oral anticoagulation therapy” [14]. Subsequently, pharmacological interactions were analyzed through a literature review. The following potentially relevant interactions were identified: (1) patient on omeprazole 40 mg/day. The literature indicates inhibition of hepatic metabolism of the anticoagulant when administered with omeprazole [15]. The risk of bleeding is lower with pantoprazole, the recommended proton pump inhibitor in patients treated with acenocoumarol [16] (equivalent dose: 20 mg of omeprazole corresponds to 40 mg of pantoprazole, 40 mg of omeprazole is replaced by 40 mg of pantoprazole, except in Zollinger–Ellison syndrome) [17]. (2) Patient on allopurinol 300 mg/day. Allopurinol may potentiate the anticoagulant effect by inhibiting the metabolism of acenocoumarol in some patients [18]. This interaction is infrequent, but when it occurs, it can be severe. INR variations may be observed, with changes in treatment and/or adherence problems. (3) Patient on paracetamol 1g, as needed. Paracetamol can alter the response to VKAs, although its effect is relatively small. As a precaution, the paracetamol dose should be limited to 2 g/day, administered for no more than 7 days. If the dose exceeds 2 g/day or the treatment duration exceeds 7 days, the INR should be rigorously monitored. Scientific evidence has shown that the use of paracetamol at doses exceeding 2.5 g per week is associated with an increased risk of an INR > 6 [19].

The patient’s relationship with vitamin K-rich foods in their diet was also analyzed using a validated test for vitamin K use in anticoagulant-treated patients [21]. The patient did not correctly identify foods with a high vitamin K content, nor was he aware of the influence of dietary habits on his anticoagulant treatment. The patient showed significant variations in the consumption of these foods in their diet. An educational intervention was conducted to encourage the patient to incorporate these foods into their diet.

The findings were compiled in a report to provide the physician with comprehensive information. Additionally, a crucial aspect of the project involved direct communication between the pharmacist and the prescribing physician. Thanks to the report’s preparation and the direct communication between the pharmacist and the physician, the physician promptly accepted the recommendations.

After implementation of the pharmaceutical interventions and treatment modifications by the prescribing physician, the Time in Therapeutic Range (TTR) was analyzed three months post-intervention. It had increased from 56.1% to 100%, indicating that the patient achieved an appropriate TTR for the effectiveness and safety of the pharmacological treatment.

## 3. Discussion

Identifying patients who may not be suitable candidates for VKA treatment allows efforts to be focused on those with optimal clinical and genetic characteristics for maintaining a stable INR. Genetic information has been shown to account for approximately 40% of the variability in the INR among patients undergoing VKA anticoagulant treatment [22]. In this case report, we demonstrate how pharmaceutical interventions in patients being treated with VKAs, particularly those without clinical or genetic variables related to poor INR control (i.e., SAMe-TT_2_R_2_ score < 2 and no relevant genetic mutations), can enhance the effectiveness and safety of this lower-cost drug, with more extensive usage and experience compared to other therapeutic alternatives like DOACs.

Despite the optimal clinical and genetic characteristics of this patient, the initial TTR was 52%, indicating the ineffectiveness of VKA treatments. Through the interview process (Table 1), it was identified that the patient faced challenges in terms of adherence and knowledge concerning both the treatment and vitamin K-rich foods. Furthermore, there were some drug interactions, and the prescribing physician was given proposed changes within the same therapeutic group, which were accepted.

Regarding adherence and knowledge, the patient in the case report exhibited unintentional non-adherence and lacked knowledge of proper medication use (Table 1). While the patient understood the medication’s indication and dosage, as well as the associated risks, he had insufficient knowledge about usage recommendations in case of missed doses and potential interactions with other drugs and/or foods (Table 4). Other studies have highlighted the limited knowledge among patients regarding VKA treatment. In a descriptive study [23], it was revealed that 40% of the 150 patients included in the sample did not comprehend the meaning of the term INR, and 81.3% were unaware of the optimal values. The majority of patients (74.0%) did not recognize interactions between warfarin and foods, drugs (aspirin or nonsteroidal anti-inflammatory drugs), alcohol, and over-the-counter products (herbal supplements and dietary items). Additionally, 84.7% lacked knowledge about dietary precautions. Concerning knowledge about INR, on average, 62% were unaware of the risks related to values outside the established range, and 40.7% did not understand the meaning of the term INR. Only 6.0% achieved a satisfactory score of ≥75%. In another observational study [24] conducted on 68 anticoagulated patients with VKAs, non-adherence to the treatment was identified as one of the primary reasons for treatment failure regarding anticoagulation with VKAs. Knowledge among these patients was also limited, with over a third unaware of the target INR for their specific condition, over half unaware of the emergency INR value, and a quarter not informed or not remembering the total duration of VKA treatment for their specific case.

Multiple studies in the literature have shown that adequate adherence and knowledge are essential for the optimal management of anticoagulation and the prevention of complications, whether hemorrhagic or embolic. In a pre-test–post-test study [25] conducted on 52 patients with atrial fibrillation and an average TTR of 38.8%, the impact of pharmaceutical care on treatment adherence was analyzed using the SMAQ (Simplified Medication Adherence Questionnaire). A 27% improvement was observed at the beginning of the treatment, rising to 85% at the end (*p* < 0.001). The TTR significantly improved from 29.03% (initial) to 46.13% after the intervention (*p* < 0.001). The observed improvement in the TTR was attributed to better treatment adherence resulting from pharmaceutical education. In another prospective, multicenter, open-label, and randomized study (the EDUCAVK project) [26], a pharmaceutical intervention consisting of a 30 min educational session on oral anticoagulation was conducted. When comparing the control and intervention groups, the average knowledge score was higher in the intervention group (13.9 vs. 12.4 points) (*p* = 0.08). It was observed that, at 3 months, the rate of hemorrhagic and thromboembolic complications decreased from 10.6% to 3.1% in the intervention group (*p* < 0.01).

Regarding the consumption of vitamin K-rich foods, the patient in the case report was unaware of which foods were rich in this vitamin and did not pay attention to incorporating these foods into their diet. As is already known, nutrition and diet can affect VKA treatment by promoting coagulation and reducing the efficacy of VKA. Although available evidence [27,28] does not support restricting vitamin K consumption while receiving warfarin treatment, on the contrary, it recommends maintaining a stable and unrestricted intake of vitamin K-rich foods. This approach prevents significant variations in consumption and response to anticoagulant treatment, thereby maintaining a more stable INR. In this regard, it seems essential to increase patients’ knowledge regarding the consumption of these foods in their diet.

However, in addition to the clear importance of adherence and knowledge for the proper use of medications, especially when they have a narrow therapeutic margin, as is the case with VKAs, other factors, such as pharmacological interactions, are highly relevant for medications with these characteristics. In the presented patient, three pharmacological interactions were identified that could potentially alter the stability of the INR: omeprazole, allopurinol, and paracetamol. Thanks to the implementation of a direct communication pathway with the prescribing physician, the replacement of omeprazole with pantoprazole was proposed and accepted. The use of VKAs with proton pump inhibitors (PPIs) can increase the risk of bleeding. In a retrospective observational study [29] conducted over five years on 126 patients treated with VKAs and PPIs, the risk was significantly higher for patients receiving omeprazole and lower for those receiving pantoprazole (*p* < 0.01). This could be attributed to the fact that all PPIs are metabolized by hepatic enzymes from the CYP group, but there are variations in the interaction potential among different PPIs. Omeprazole has the highest potential to alter metabolic activity at the CYP enzyme level, while it is lower with other PPIs, such as pantoprazole [30].

A moderate interaction between allopurinol and VKA was also identified. Allopurinol can inhibit the metabolism of VKA [31], possibly enhancing its anticoagulant effect. The risk of minor bleeding could increase by 19 times [32], making it essential to monitor the INR more closely. Therefore, close monitoring of this interaction was recommended, and patient adherence, especially to medications interacting with acenocoumarol, was reinforced, emphasizing that irregularities in taking these medications could increase INR variability.

Some of the strengths of this case report lie in its multidisciplinary approach to addressing the problem, exploring and researching areas where patients can improve medication outcomes, such as knowledge and adherence factors. Additionally, it underscores the importance of generating evidence on the utility of pharmaceutical interventions. This case also demonstrates the effectiveness of VKA, as long as adequate control over all the interacting variables is achieved.

However, the main limitation of this clinical case report is its low reproducibility, since the use of genetic tools in daily clinical practice is not common. Furthermore, it could entail an additional expense that may not be easily affordable for all patients, especially if it is not covered by the public healthcare system. Nonetheless, this type of evidence is crucial at present, as many countries are beginning to integrate pharmacogenetics into their healthcare systems, not only in research but also in clinical practice.

## 4. Conclusions

The use of predictive tools for poor INR control can help select patients who can effectively and safely use VKAs, by means of pharmaceutical interventions tailored to the patient’s needs, in collaboration with the prescribing physician. In this patient, we have been able to verify that this strategy for caring for anticoagulated patients is feasible and yields optimal results.

## Figures and Tables

**Table 1:** Procedure of the pharmaceutical intervention. General practitioner (GP); practice change facilitator (PCF); Time in Therapeutic Range (TTR).

**First visit**	The pharmacist performs a systematic interview with 16 pre-designed open-ended questions about the patient’s health problems and treatments. Genetic information, SAMe-TT2R2.	
**Second visit**	The pharmacist performs an educational session, presents the intervention proposal, and material delivery, with the objective of improving patient adherence.	
	**Problem**	**Recommended educational technique**
	Non-adherence	Intentional: motivational interview, diary about an activity Involuntary: SPD, calendars, alarms…
	Interactions	Practical information, action measures, simulation techniques
	Diet and phytotherapy	Vitamin K content. Establish a contract, journal about an activity. Information about other types of interactions.
**Third visit**	The pharmacist checks the result of the interventions and sends a report summarizing the main outputs from the performance and provides recommendations to the GP. Final data collection by PCF: TTR, hemorrhagic/thrombotic events, knowledge, adherence, satisfaction, and quality of life questionnaire treatment variables, acceptance of pharmaceutical interventions by GP and patient.	

**Table 2:** Baseline characteristics of the patient.

Age (years)	84
Gender	Male
Height (cm)	150
Weight (kg)	70
BMI	31.11
Race	Caucasian
Education level	No studies
Number of cigarettes per day	0
Daily vitamin K intake (g)	15
Mobility	No issues
Personal hygiene	No issues
Daily activity	No issues
Pain/discomfort	No
Alcohol consumption (drinks/day)	1
Physical activity	No
Indication for VKA	Atrial fibrillation
Pre-TTR (%)	56.1
Post-TTR (%)	100
Number of medications	11

Note: body mass index (BMI).

**Table 3:** Pharmacogenetic information on the patient.

Patient Genotype	Functional Status	Information
CYP2C9 *1/*1	Normal metabolizer	Not associated with an increased risk of bleeding. It may be necessary to check the INR more frequently after initiating or modifying drugs that may potentially interact.
VKORC1 *1/*2	Decreased function	It is associated with increased sensitivity to coumarin anticoagulants and a lower dosage requirement. These patients may need more frequent INR monitoring.
CYP4F2 *1/*1	Normal function	Not associated with increased sensitivity to coumarin anticoagulants and a lower dosage requirement.

Note: International Normalized Ratio (INR).

**Table 4:** Issues identified in regard to the patient and the measures implemented by the pharmacist.

Identified Problem	Measures Implemented by the Community Pharmacist
Unintentional non-adherence	Educational intervention: information about prescribed medications; Information about the importance of adherence and compliance with the medical regimen for proper control; Provision of a medication schedule; Training on the correct use of inhalers.
Interaction with omeprazole	Propose replacement with pantoprazole (intervention accepted by the prescribing physician).
Interaction with allopurinol	Monitoring INR to control potential variability; Educational intervention on the need to be consistent in the timing of allopurinol and VKA administration to minimize INR variability.
Interaction with acetaminophen	Educational intervention: inform the patient that the dose should not exceed 2 g/day and should not be taken for periods longer than 7 days without medical supervision.
Vitamin K intake	Educational intervention: information is provided about the vitamin K content in foods, recommendations for a balanced intake of these foods in the patient’s diet, and supplementary educational materials provided. Vitamin K, present in some foods and dietary supplements, is involved in hemostasis (blood clotting) [20].

## Data Availability

The data that support the findings of this study are available on request from the author, Ana María Sáez-Benito.
